# Supplementary figures and images for: Genetic depletion studies inform receptor usage by virulent hantaviruses in human endothelial cells
Source: eLife. 2021 Jul 6;10:e69708. doi: 10.7554/eLife.69708 (PMC8263056; doi:10.7554/eLife.69708)

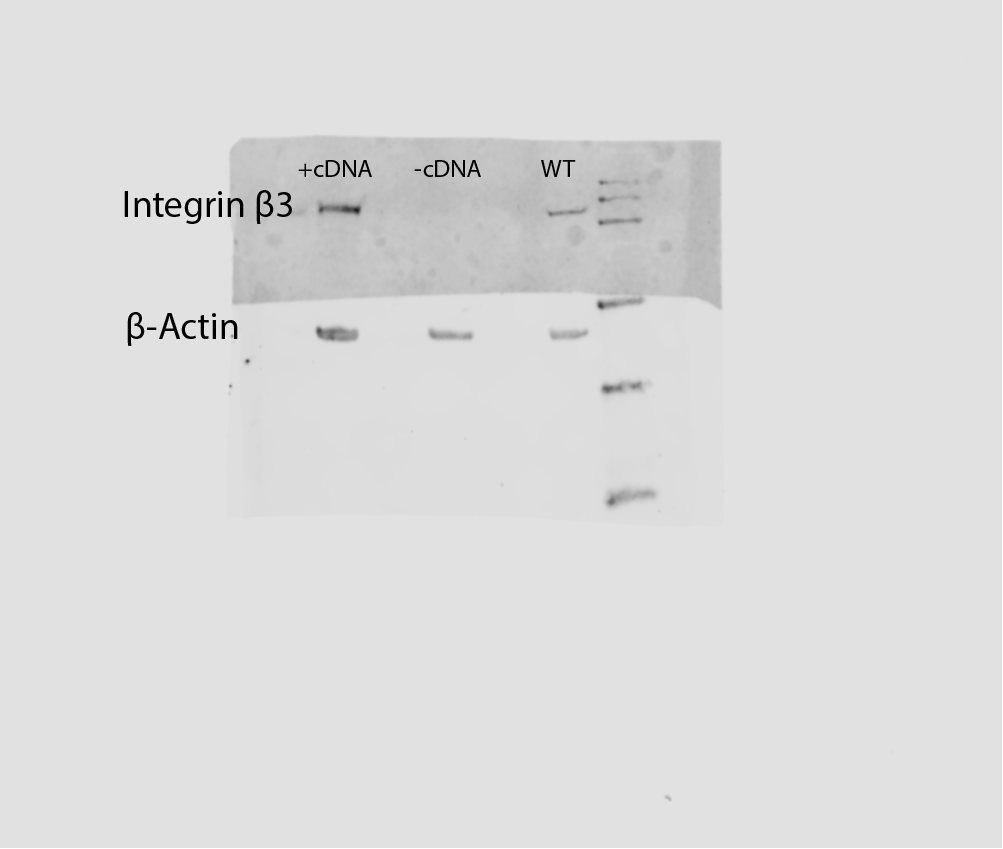

Supplement: Figure 1—source data 1. [file elife-69708-fig1-data1.zip › WB_original_itgb3_labelled.png]

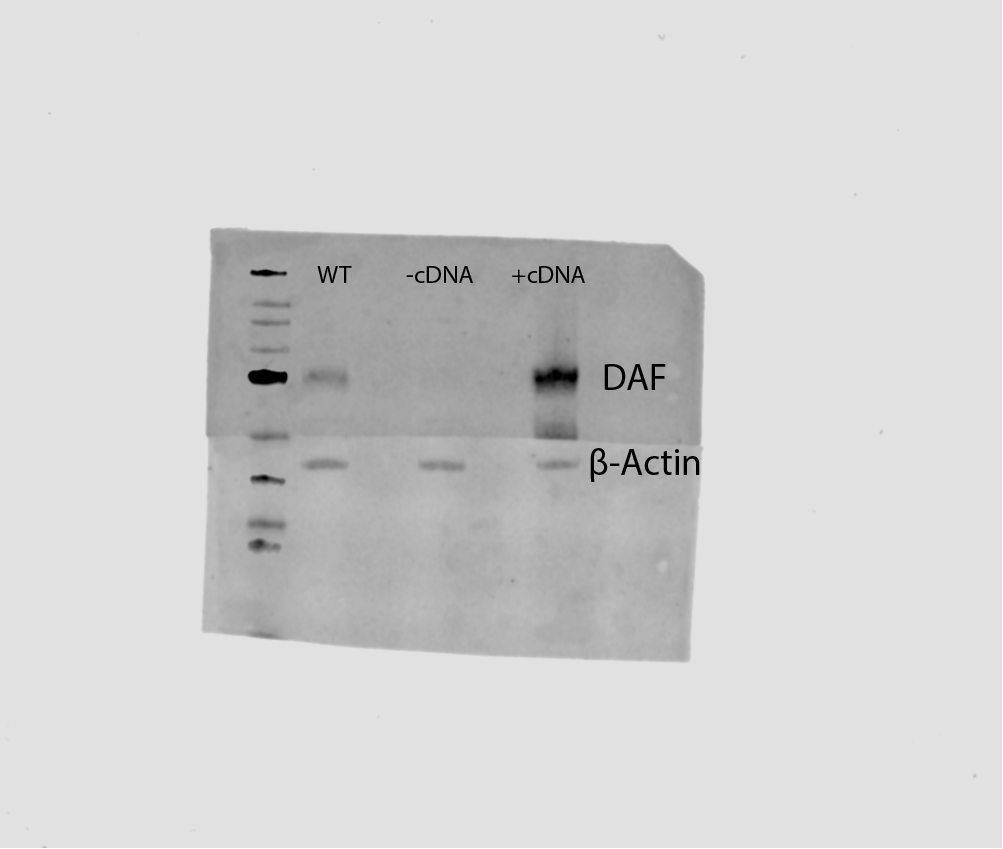

Supplement: Figure 1—source data 1. [file elife-69708-fig1-data1.zip › WB_original_DAF_labelled.png]

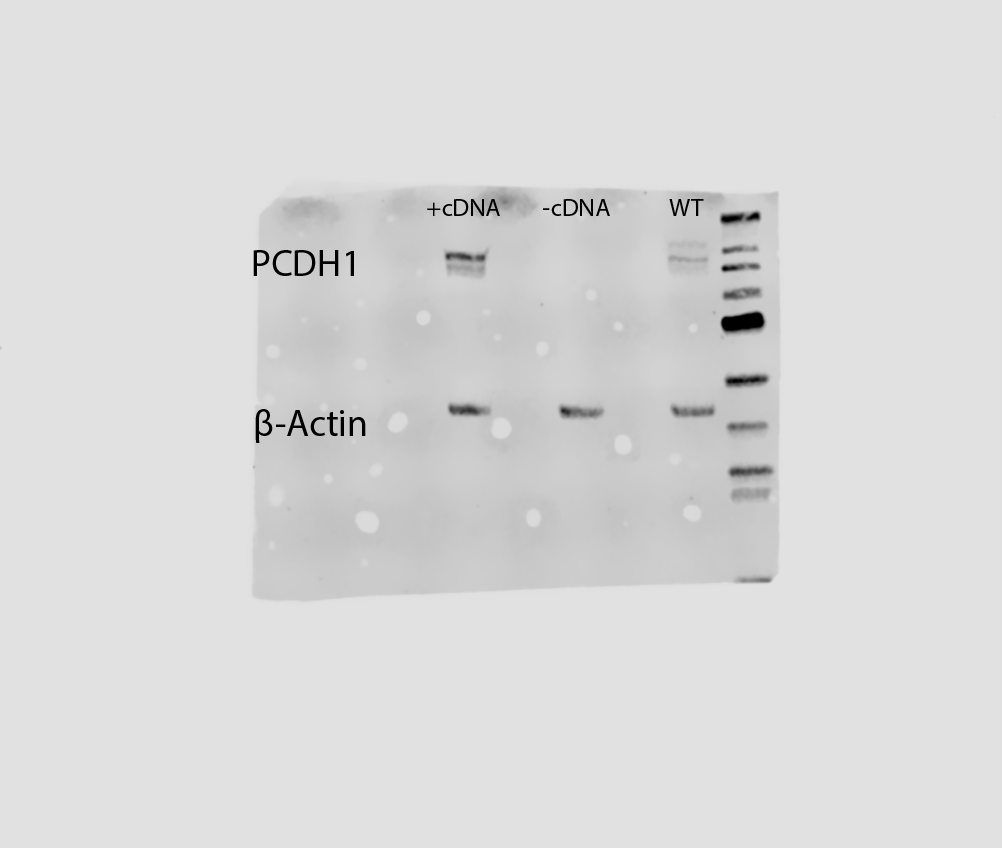

Supplement: Figure 1—source data 1. [file elife-69708-fig1-data1.zip › WB_original_pcdh1_labelled.png]

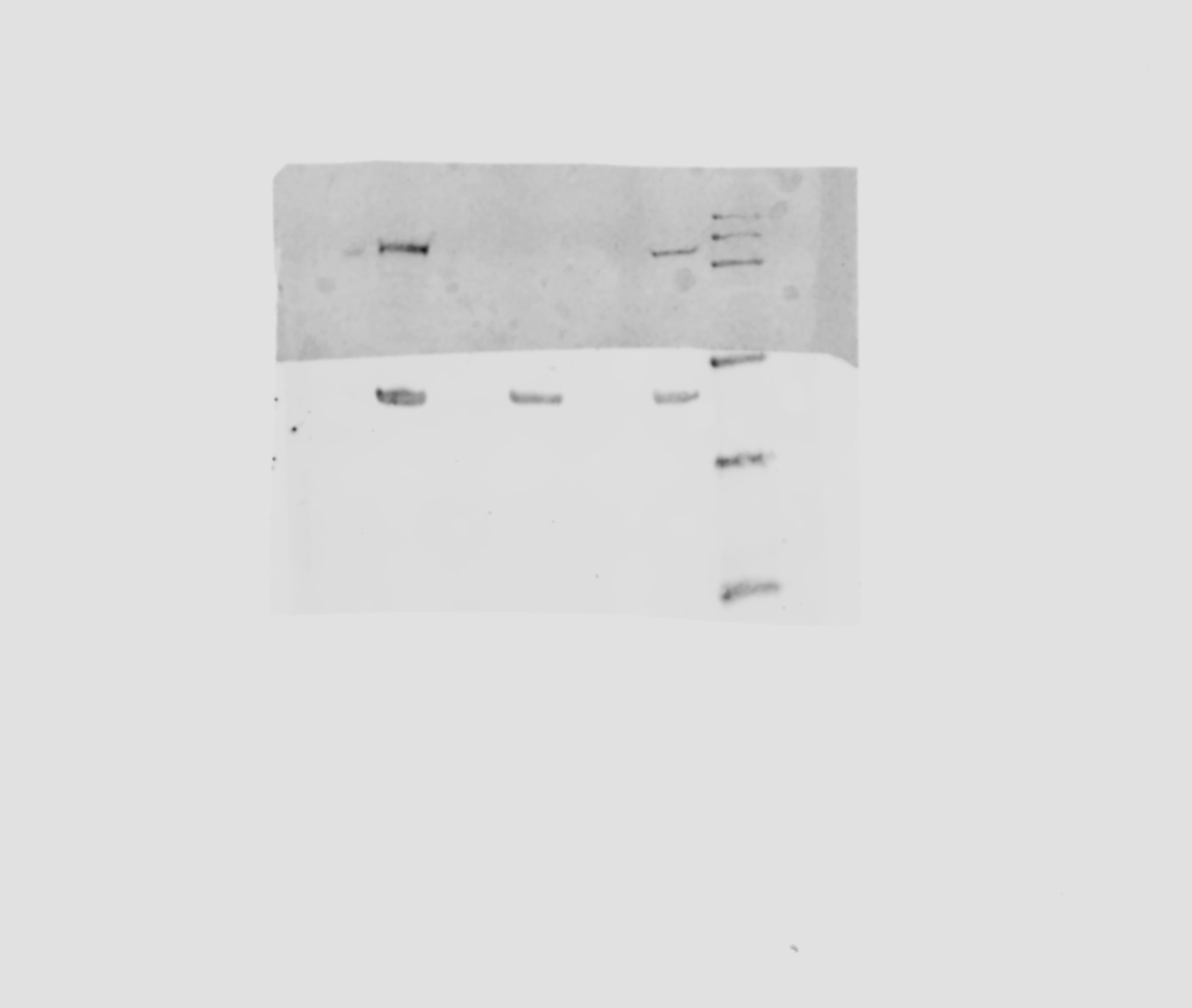

Supplement: Figure 1—source data 1. [file elife-69708-fig1-data1.zip › WB_original_itgb3.tif]

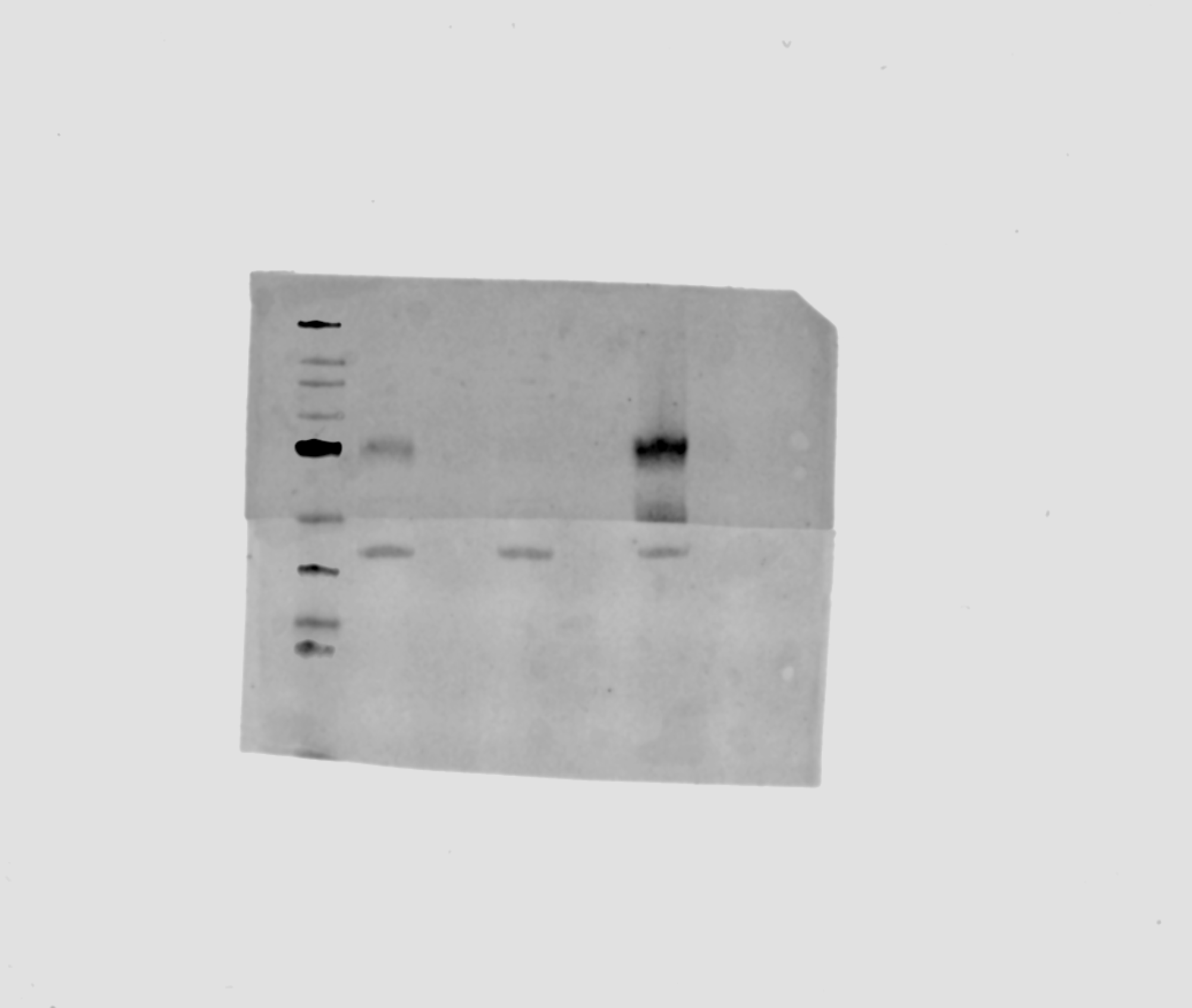

Supplement: Figure 1—source data 1. [file elife-69708-fig1-data1.zip › WB_original_daf.tif]

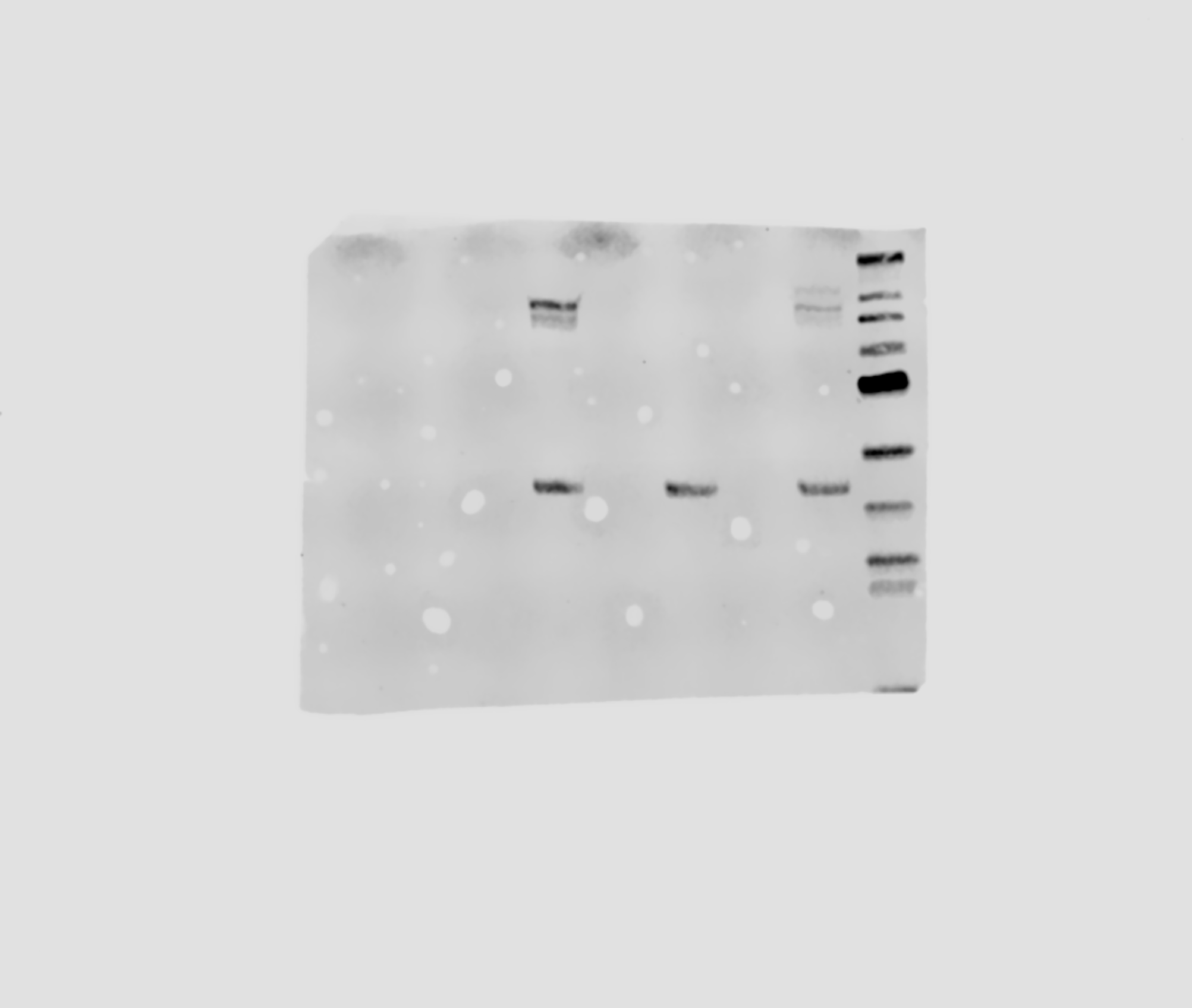

Supplement: Figure 1—source data 1. [file elife-69708-fig1-data1.zip › WB_original_pcdh1.tif]
